# Prevalence and impact of grief among student veterinary nurses: A cross‐sectional study

**DOI:** 10.1002/vetr.5596

**Published:** 2025-06-09

**Authors:** Freya Wheatley, Evie Yon

**Affiliations:** ^1^ Highcroft Veterinary Group Hailsham UK; ^2^ Royal Veterinary College Hatfield UK

## Abstract

**Background:**

The veterinary profession exposes employees to potentially multiple deaths a day. Student veterinary nurses can experience patient loss from their first year of training. This study explores grief in student veterinary nurses in the UK and examines if there is an association between their Adult Attitude to Grief Scale (AAGS) scores and their recognition of grief.

**Methods:**

This cross‐sectional study used a survey incorporating the AAGS to determine whether the participants were suffering from grief. They were then asked whether they felt supported by both their academic institution and training practice.

**Results:**

Survey responses indicated that all of the 330 student veterinary nurses participating in the study had suffered from grief, yet only 89.1% (294/330) believed that they had experienced grief. Furthermore, 83% (274/330) felt supported by their institution, and 82.4% (272/330) felt supported by their practice. There was a statistically significant relationship between the recognition of grief and AAGS scores.

**Limitations:**

This was a student project, and financial and time constraints led to smaller sample sizing, which meant that the study was underpowered for statistical analysis.

**Conclusion:**

This study's findings indicate that student veterinary nurses are affected by grief and need further training and support. This small population of the veterinary industry could reflect a wider issue within veterinary professionals.

## INTRODUCTION

### Grief and disenfranchised grief

Grief is ‘the anguish experienced after significant loss’,^1^ which can cause depression, numbness, detachment, feeling overwhelmed and changes to the body and normal behaviour.^2^ This can manifest in many ways, including disenfranchised grief (i.e., unacknowledged grief and isolated grieving due to societal norms^3^).

Disenfranchised grief is a researched topic in both veterinary and human medicine, where healthcare workers are presented as the ‘forgotten mourners’.^4^ Disenfranchised grief can develop into compassion fatigue if not properly processed.^5^ Marton et al.^3^ explored disenfranchised grief in animal care workers and how the loss of the animals in their care is similar to losing their own pet. They further go on to state how there was a distinct lack of understanding from their support systems, which further increases the risk of disenfranchised grief developing.^6^ Harvey and Cameron^7^ elaborated on how 82% of veterinary nurses suffer from compassion fatigue; the exhaustion stemming from potentially traumatic situations, such as euthanasia. These studies highlight the increased risk of disenfranchised grief and compassion fatigue, concluding that more support is needed for veterinary professionals when dealing with their grief.

### Veterinary nurses’ role in supporting clients through patient death

Experiencing euthanasia and patient death is an unfortunate part of the veterinary nursing role. Communication with the client and the veterinary team about the euthanasia process is an important factor to ensure a positive euthanasia.^8^ There is a lot of research into how veterinary nurses can provide a good level of care for their clients. This includes following standard operating procedures^9^ to help create memorial pieces, such as a print of their pet's paws or hair clippings.^10^ Lee^8^ stated how veterinary nurses can provide a great amount of support following euthanasia, including body care options, keepsakes and follow‐up bereavement support. MacDonald^11^ further explained how veterinary nurses must support the client throughout the patient's life, including end‐of‐life and palliative care, where veterinary nurses take on an emotionally supportive role. Veterinary nurses can help the client process their grief positively by helping recount memories^8^; this can be beneficial when paired with follow‐up communication. By being involved in the end‐of‐life process of their patients, veterinary nurses are at a higher risk of experiencing their own grief.^12^ MacDonald^11^ further recognised that veterinary nurses’ mental wellbeing can be affected by euthanasia. Veterinary nurses may have built strong relationships with clients and patients prior to the patient's death, which can therefore be an emotional experience for the veterinary nurse.^10^, ^13^ Although recorded in the literature as a possible issue, there is currently a limited evidence base presenting findings about how patient loss affects the veterinary nurse. This could indicate that the prevalence of grief due to patient loss is underestimated, with veterinary nurses left unsupported. It is widely recognised that veterinary nursing is a challenging and demanding profession.^7^ Patient loss forms a significant part of the role, and without support, not only is mental wellbeing affected, but a veterinary nurse's ability to support clients through pet loss may also be affected.^14^

### Grief and veterinary nurses

It is recognised that veterinary nurses experience their own grief when it comes to the death of their patients.^10^ When grief is not processed healthily and continued grief is felt through multiple patient losses, it can lead to more serious mental health complications such as depression and suicidal ideation.^15^ Deacon and Brough^14^ found that exposure to patient suffering and death caused an increase in psychological distress, leading to its recognition as a health and safety risk within veterinary practice.^16^ The veterinary profession is increasingly aware of the importance of prioritising the preservation of mental wellbeing.^12^ It has been reported that veterinary nurses are more likely to provide a social worker‐like role for clients, which can increase the risk of occupational stress as it is not a trained part of nursing.^17^ This is significant because, in the Royal College of Veterinary Surgeons (RCVS) survey of the veterinary nursing profession, veterinary nurses had the lowest wellbeing score compared to other roles within clinical practice.^18^ A case study strategy from Ballantyne^19^ aimed to help newly graduated veterinary nurses cope with the grief they may experience at work but stressed that the emotional experience needed to help cope is a skill that is learned over time. This leaves students and newly graduated veterinary nurses vulnerable to grief and other conditions that affect wellbeing.^7^ This cumulative impact could influence the ability to work effectively, increasing the chance of leaving the profession.^20^

### Knowledge gap

The impact of patient loss is somewhat reported in veterinary medicine, yet not enough to bring widespread awareness and change.^16^ Despite the topic gaining traction within the veterinary industry, there is still a need for more research^21^ to affect positive change to protect the wellbeing of veterinary professionals.^22^ For veterinary nurses, both registered and student, the research further dwindles, focusing rather on the clients’ grief than the emotional load placed on veterinary nurses.^23^ This study therefore aimed to contribute an insight into the prevalence of grief in the UK student veterinary nurse population.

## MATERIALS AND METHODS

Data for this study were collected between January and early February 2024. This cross‐sectional study used a survey to establish the prevalence and awareness of grief in the student veterinary nurse population in the UK.

### Participants

The target population was all student veterinary nurses enrolled with the RCVS, including both degree (Foundation and Bachelor's) and diploma routes of training. Exposure to patient death and euthanasia is almost certain at the student level, as a minimum of 1800 hours of placement in an RCVS‐accredited training practice is required to be eligible to qualify and enter the RCVS Register for Veterinary Nurses. Additionally, student veterinary nurses must have experienced at least one patient death during their training to be eligible to enter the Register. Student veterinary nurses were exclusively chosen to identify whether grief and its associated issues manifested in training rather than in the workplace post‐registration.

The participants were recruited via social media. Student nursing groups on various platforms, including Facebook, were used to promote and share the survey, which relied on snowball sampling. Course directors for veterinary nursing programmes at 30 institutions were contacted, with 18 willing to share the survey with their students. Institutions were contacted again if they had not responded after 3 weeks. Peers of the primary researcher (F.W.) were also contacted to share the survey with student veterinary nurses within their network. On social media, the survey was shared twice by the primary researcher over the 5‐week period it was open.

In 2023, a total of 1007 student veterinary nurses qualified and entered the RCVS Register for Veterinary Nurses, resulting in an approximate target population of 3027^24^ to include student veterinary nurses in each of the three years of training. With a statistical calculator,^25^ the estimated population was entered with a 95% confidence level and a 5% margin for error. This power calculation indicated that a sample size of 341 participants was required to be able to calculate statistical significance.

### Survey design

The survey opened with questions to ascertain the number of patient deaths experienced and self‐identification of experienced grief. The main body of the survey was primarily designed using the Adult Attitude to Grief scale (AAGS),^26^ which is a validated nine‐question scale that considers how the participant may be coping with grief and their vulnerability to their grief. The questions are grouped into three categories: balanced, controlled and overwhelmed. These three categories reflect the range of responses to loss and allow the participants' grief to be articulated following the three main grief‐processing styles.^26^ Balanced represents acknowledgement of grief and processing of feelings healthily. Controlled is where the participant suppresses their emotions and seeks to control their distress. Overwhelmed represents the participant being drastically impacted by their grief, such that it affects their day‐to‐day life. The total scores for the AAGS range from 0 to 36 and can be categorised into three groups: low, less than 20; high, 21‒23; and severe, greater than 24. This scale was used because it can identify the prevalence of grief and how vulnerable the participants are.^26^ The vulnerability aspect of this scale allowed for this study to explore the severity of grief experienced by participants, which could present data on whether students are feeling supported by their institution and practice.

Following the AAGS style questions, the survey gathered information about the perceived level of support from institutions and placement practices, route of training, year of study and type/areas of practice experienced. The AAGS‐style questions were scale‐like questions ranging from strongly disagree to strongly agree. The support questions were binary (yes/no) questions, and the demographic questions were either short‐answer questions or multiple choice.

### Data analysis

The survey data were analysed using descriptive statistics. In addition, the relationship between participants' AAGS scores and their recognition of experiencing grief was assessed using an independent *t*‐test with significance set at a *p*‐value of less than 0.05. Data analysis was conducted using SPSS.

## RESULTS

There were 342 responses to the survey; 12 responses had to be excluded due to incomplete questions, leaving 330 included responses. The participants consisted of 73 degree students (22.1%) and 257 diploma students (77.9%). When dealing with feelings of grief, 83% (*n* = 274) felt supported by their institution and 82.4% (*n* = 272) felt supported by their practice. Of the degree students, 32.9% (*n* = 24/73) did not feel supported by their institution, with 12.5% (32/257) of diploma students feeling the same. Some degree students (15.1%; 11/73) and diploma students (18.3%; 47/257) did not feel supported by their training practice. Of the degree and diploma students, 8.2% (6/73) and 5.1% (13/257), respectively, felt unsupported by both their institution and practice when dealing with their grief.

Of the 330 participants, 118 (35.8%) were first‐year students, 133 (40.3%) were second‐year students and 79 (23.9%) were third‐year students. Over half (59.4%; 196/330) of the participants had worked in general practice, 11.2% (37/330) had worked in non‐referral veterinary hospitals and 6.4% (21/330) had worked in referral hospitals. Fifty‐eight participants (17.6%) had worked in two types of practice and 18 (5.5%) had worked in all three types.

Overall, 89.1% (294/330) of participants felt that they had experienced feelings of grief. The number of patient deaths experienced was heavily weighted, with 79.7% (263/330) of participants experiencing more than 10 patient deaths; meanwhile, 10.3% (34/330) reported experiencing less than five deaths and 10% (33/330) reported experiencing 5‒10 deaths. No participants had an AAGS score of 0, meaning that they had all experienced grief at some point during their training. Overall, 87.3% (288/330) of participants had low AAGS scores, 12.1% (40/330) had high scores and 0.6% (2/330) had severe scores. The lowest AAGS score reported was 6 and the highest was 27. The histogram of the AAGS scores was normally distributed, with a mean of 16.69 and a standard deviation of 3.26. Out of the 288 participants with low scores, 11.8% (34/288) did not think that they had experienced feelings of grief. In the high score group, 5% (2/40) felt that they had not experienced feelings of grief. All (2/2) of the severe scoring participants said yes to experiencing feelings of grief. Those who felt that they had experienced grief had a higher mean score (16.92) than those who felt that they had not experienced grief (14.83) (*p* < 0.001).

Out of the AAGS balanced, overwhelmed and controlled categories, the controlled category had the highest average score (8.32), followed by the overwhelmed category (5.14) and the balanced category (3.23). The controlled category having the highest average score means that participants have an increased risk of suppressing their feelings of grief and have greater avoidance of expressing emotions related to grief. These factors have been shown to have a detrimental effect on mental health.^26^ Furthermore, the next highest scoring category was overwhelmed, with those participants experiencing high levels of grief that affect their daily life. The lowest average was balanced, meaning that participants are less likely to be processing grief adaptively, even if they acknowledge their experience of grief.

## DISCUSSION

### Prevalence of grief

All of the participants in this study experienced grief during their training. This was expected as grief is a completely natural response to loss, and student veterinary nurses are required to experience euthanasia as part of their training in supporting clients through the loss of their pet.^27^ This study found that 89.7% of the students surveyed had experienced more than five patient deaths, potentially identifying an underestimation of the frequency of euthanasia and patient loss that students are experiencing. This could indicate that student veterinary nurses are more involved in practice than initially thought. With student veterinary nurses being more involved with patients, it allows a bond to form between the patient and veterinary nurse.^28^ Closeness increases the chance of more severe grief,^29^ increasing the chance of developing into more severe mental health issues if it is not dealt with healthily, potentially affecting the ability to work. With more involvement comes more opportunities; however, students felt that they needed more support and training in bereavement situations.^30^ This further introduces the need for more training to help improve and protect wellbeing,^30^ especially as it is a skill learned over time.^19^ Furthermore, if students are not coping healthily with their grief, it could be brought into the workplace, affecting their training and performance.

Many students had low grief vulnerability scores (87.3%), yet there was a higher average score in the controlled category of questions, indicating a decreased acknowledgement of distress and an increased self‐reliance,^26^ which is known to lead to disenfranchised grief.^3^ This is relevant to veterinary nurses, who often spend multiple days nursing and bonding with the patient^23^ and are then the ones to be present for their death and comfort the owner. The veterinary nurse has to suppress their emotions^31^ and is usually too busy attending to other duties^20^ to take time to acknowledge and process those feelings of grief. The lack of time to acknowledge any emotions felt further pushes veterinary nurses towards suffering from disenfranchised grief. This can contribute heavily to compassion fatigue and burnout.^6^ When the affected continue to ‘push through’ more problems can arise, which can compromise their ability to work.^7^ Additionally, if this continues post‐registration, their ongoing issues with grief may be exacerbated due to increased responsibility and involvement within practice. This may negatively impact individual veterinary nurses and, in turn, may reduce the effectiveness of the veterinary team.

### Recognition of grief

The significant relationship between AAGS score and whether the participant had felt they had experienced grief indicates that students have some recognition of when they are experiencing grief. This could also indicate that students must be more severely vulnerable to grief to recognise the signs, as the higher the scores the more students recognised their grief. This could further be detrimental to their mental health if students are more vulnerable to their grief and do not know how to properly process these emotions in a healthy manner.

Overall, 10.9% of participants recorded that they had not experienced grief, even though they had experienced patient death. This could indicate that some students do not know what the symptoms of grief are and therefore do not recognise them, indicating that students may need more training on grief. More education could consequently improve mental wellbeing in later professional life, as the symptoms can be recognised and managed appropriately. Veterinary nurses are exposed to the same patient suffering and loss that veterinary surgeons are, and this was found to have a positive correlation to psychological strain and burnout.^14^ Student veterinary nurses are being exposed to euthanasia and death within their first year of training, with potentially no experience prior to dealing with these situations and the emotions that accompany them, impacting their mental wellbeing.^22^ Again, the strategy from Ballantyne^19^ stresses that the emotional experience needed to help cope is a skill that is learned over time, increasing the student veterinary nurse's vulnerability to grief and other conditions that affect wellbeing.^7^ This could be alleviated with support from institutions and practices.

### Support

While most students felt supported (83% by their institution and 82.4% by their practice), there were some who felt unsupported. Degree students felt less supported by their institution than diploma students; however, diploma students spend less time at their institutions compared with degree students, which could mean that they have to rely on them less and consequently are unsatisfied less often.^32^ The same theory could apply to diploma students and how they feel more unsupported by their practice. Degree students spend less time in practice; therefore, they could have less of a relationship with the employers and would rather go to their institution for support.^32^ A higher number of degree students felt unsupported by both institutions and practices (8.2% [6/73] of degree students and 5.1% [13/257] of diploma students). For both degree and diploma students, studies show that those who feel that they are unsupported are more likely to develop disenfranchised grief,^3^ which harms their wellbeing because their grief is unacknowledged. This further indicates that there should be more training on grief from institutions, which could allow for better understanding and empathy regarding the symptoms of grief.

#### Health and wellbeing support

To protect wellbeing within practice, there needs to be more support from management and employers, which is what Deacon and Brough^14^ concluded in their study. Management personnel are responsible for providing safe and manageable workplaces where staff thrive rather than drown. The industry is understaffed, with a reported 19.5% wanting to leave the veterinary nursing profession.^20^ With a better approach to preserving good mental wellbeing, job satisfaction, and thus staff retention, is likely to improve.^33^ Patient loss is not an easy process for owners or veterinary professionals.^34^ For most, they would have spent a considerable period caring for and treating an animal prior to presenting euthanasia as an option.^13^ Veterinary surgeons must make ethical decisions that can cause moral stress, which negatively affects mental wellbeing.^35^ Veterinary nurses are involved in ethical decision making just as much as veterinary surgeons,^24^ and veterinary nurses have been found to suffer from moral stress more severely than veterinary surgeons.^36^ Moral stress further affects mental health, causing feelings of frustration, guilt and failure.^35^ This can lead to more serious mental health complications, such as depression and suicidal ideation.^15^ A good team can build a support system using the correct protocol,^12^ such as debriefing, which can assist in reducing the negative impacts of stressful situations, including patient loss. With 87.6% of veterinary surgeons experiencing feelings of grief due to the loss of a patient,^16^ specific support is needed to aid the processing of grief. The negative psychological impacts that unprocessed grief can cause^4^ are important to address. Women are more likely to suffer from more complicated grief more frequently than men.^37^ With 96.8% of veterinary nurses being female,^18^ and female health professionals being at higher risk of suicide,^38^ specific support is needed to prevent this from happening.

Based on the findings presented in this study, the authors recommend that greater training surrounding the recognition and management of grief should be embedded within veterinary nursing curricula. In addition to this, grief support should be made readily available through educational institutions, and if this is not possible, explicit signposting to available external support should be ensured.

### Limitations

Due to this study being a student research project with limited time and finances, there was less time to collect data. This meant that completed surveys were received from fewer participants than specified by the sample size calculation (330 versus 341). As such, the statistical analyses were likely underpowered. The results should therefore be interpreted cautiously.

## CONCLUSION

This study concludes that grief is prevalent among student veterinary nurses, with some of them not recognising when they are experiencing grief. This can be detrimental to their mental wellbeing. While most feel supported, institutions should provide more education to prepare and support students experiencing grief during the training process. While this study was limited to student veterinary nurses only, the findings could indicate a more widespread problem within the veterinary industry, especially with the high dissatisfaction rates causing many to leave clinical practice. Greater education about and access to support for grief are necessary to ensure that healthy practices are being undertaken. Not only would this benefit individual members of the veterinary nursing profession, but also the professional workforce as a whole.

## AUTHOR CONTRIBUTIONS

Freya Wheatley devised the project and its main conceptual ideas, carried out the technical aspects of the research and formulated the discussion. Freya initially wrote up the project as a dissertation with Evie Yon supervising. Evie guided the project and aided in interpreting and applying the results to the relevant areas of practice, in addition to contributing to the writing of the final manuscript for publication.

## CONFLICT OF INTEREST STATEMENT

The authors declare they have no conflicts of interest. This research had no funding.

## ETHICS STATEMENT

Ethical approval for this research project was given by the Royal Veterinary College's Social Sciences Research Ethical Review Board with the URN SR2023‐ 0166.

## Data Availability

The data that support the findings of this study are available from the corresponding author upon reasonable request.
